# Evaluation of whole-genome sequence to predict drug resistance of nine anti-*tuberculosis* drugs and characterize resistance genes in clinical rifampicin-resistant *Mycobacterium tuberculosis* isolates from Ningbo, China

**DOI:** 10.3389/fpubh.2022.956171

**Published:** 2022-08-18

**Authors:** Yang Che, Yi Lin, Tianchi Yang, Tong Chen, Guoxin Sang, Qin Chen, Tianfeng He

**Affiliations:** ^1^Institute of Tuberculosis Prevention and Control, Ningbo Municipal Center for Disease Control and Prevention, Ningbo, China; ^2^Center for Health Economics, Faculty of Humanities and Social Sciences, University of Nottingham, Ningbo, China; ^3^Department of Disease Prevention and Health Promotion, Hwa Mei Hospital, University of Chinese Academy of Sciences, Ningbo, China

**Keywords:** drug resistance, drug susceptibility test, whole-genome sequencing, rifampicin-resistant tuberculosis, gene mutation

## Abstract

**Setting:**

Controlling drug-resistant *tuberculosis* in Ningbo, China.

**Objective:**

Whole-genome sequencing (WGS) has not been employed to comprehensively study *Mycobacterium tuberculosis* isolates, especially rifampicin-resistant *tuberculosis*, in Ningbo, China. Here, we aim to characterize genes involved in drug resistance in RR-TB and create a prognostic tool for successfully predicting drug resistance in patients with TB.

**Design:**

Drug resistance was predicted by WGS in a “TB-Profiler” web service after phenotypic drug susceptibility tests (DSTs) against nine anti-TB drugs among 59 clinical isolates. A comparison of consistency, sensitivity, specificity, and positive and negative predictive values between WGS and DST were carried out for each drug.

**Results:**

The sensitivities and specificities for WGS were 95.92 and 90% for isoniazid (INH), 100 and 64.1% for ethambutol (EMB), 97.37 and 100% for streptomycin (SM), 75 and 100% for amikacin (AM), 80 and 96.3%for capreomycin (CAP), 100 and 97.22% for levofloxacin (LFX), 93.33 and 90.91% for prothionamide (PTO), and 70 and 97.96% for para-aminosalicylic acid (PAS). Around 53 (89.83%) and 6 (10.17%) of the isolates belonged to lineage two (East-Asian) and lineage four (Euro-American), respectively.

**Conclusion:**

Whole-genome sequencing is a reliable method for predicting resistance to INH, RIF, EMB, SM, AM, CAP, LFX, PTO, and PAS with high consistency, sensitivity, and specificity. There was no transmission that occurred among the patients with RR-TB in Ningbo, China.

## Introduction

Rifampicin-resistant *tuberculosis* (RR-TB) is often diagnosed with genotypic or phenotypic techniques. RR-TB has become an important global health concern. Tracking RR-TB burden requires global efforts, including prevention, diagnosis, treatment, and surveillance (1). The medical treatment duration of RR-TB is longer compared with TB, which responds to drugs. However, more costly and toxic drugs may shorten the duration (2). According to the World Health Organization (WHO), ~0.46 × 10^6^ new cases of RR-TB were reported worldwide in 2019. Furthermore, 78% of these cases were resistant to multiple drugs (3). China reports 66,000 cases of RR-TB yearly and ranks second among countries with high multidrug-resistant *tuberculosis* (MDR-TB) (3).

At the 1st stage in clinics, detecting drug resistance is critical to perform optimistic medical treatment together with avoiding the occurrence and transmission of extensively drug-resistant *Mycobacterium tuberculosis* (XDR-TB) (4). Over the past decades, phenotypic susceptibility has remained the primary way of diagnosing drug-resistant TB. However, phenotypic susceptibility tests only a few drugs, and the process is not quick (~6 weeks) (5). Molecular-based drug susceptibility test (DST) has recently been conducted in clinical laboratories to detect drug resistance and TB transmission dynamics (6, 7). Reports derived from recent studies are only available to a limited number of mutations and thus most likely do not identify heteroresistance (8, 9).

Whole-genome sequencing methods based on DNA sequencing platforms to reconstruct the complete genome DNA sequence can provide high-resolution genotyping and identification (10–12). The single-chromosome genome of MTBC bacteria makes WGS techniques ideal. Using DNA data from common pathways of drug resistance, WGS helps in the prediction of drug resistance (13, 14). Recently, the ease and cost-effectiveness of WGS in predicting resistance to anti-TB drugs have been established (15–17).

To date, WGS remains poorly investigated in China, and data on RR-TB remain anemic. Here, we conducted WGS for the prediction of RR-TB resistance to multiple drugs. We carried out this study in Ningbo, China, which has a relatively high annual TB incidence. Therefore, we compared WGS with the DST of 9 anti-TB drugs. We also included lesser-known drugs such as PAS and PTO.

## Materials and methods

### Isolates from clinical samples

We randomly selected *N* = 59 RR-TB isolates from our RR-TB inventory (stored at −80°C, Ningbo CDC, 1 January 2018 and 30 December 2019).

Culture-based DST was conducted for the nine anti-TB drugs. All the isolates were selected among patients with TB who have been residents of Ningbo for at least 6 months and have tested negative for human immunodeficiency virus (HIV) and tested positive for TB in local clinics. Exclusion criteria included pregnant women, minors (age <18), and patients with severe liver or renal diseases.

### DST

Drug susceptibility tests of four first-line anti-TB drugs and five second-line drugs were carried out based on WHO recommendations (18). The drug concentrations are INH 0.2 μg /ml, RIF 40 μg/ml, EMB 2 μg/ml, SM, 4 μg/ml, LFX 2 μg/ml, AMK, 30 μg/ml, CAP 40 μg /ml, PTO 40 μg/ml, and PAS 1 μg/ml (18). H37RV strains were used as a reference for quality control.

### WSG procedure and analysis

Genomic DNA from MTB colonies scraped from an L–J medium was detected to conduct sequencing with the CTAB technique of DNA purification. Quantification of DNA was carried out using the Qubit 2.0 fluorometer (Invitrogen, Carlsbad, CA, United States). Preparations of the library were carried out according to the manufacturer's instructions (Illumina TruSeq DNA Nano Library Prep Kit). Illumina HiSeq equipment (Illumina, San Diego, CA, Untied States) was used for libraries with different indices. A 2 × 150 paired-end (PE) configuration was employed during sequencing. MTB H37RV (GenBank accession no. NC 000962.3) was used as the reference strain. Drug resistance and strain-specific signatures were exported directly from raw sequences in the “TB-Profiler” tool (https://tbdr.lshtm.ac.uk/)^19^).

### Phylogeny construction

Using the Random Accelerated Maximum Likelihood software (RAxML), a phylogenetic tree was created to evaluate evolutionary connections between 59 RR-TB strains. Single nucleotide polymorphisms (SNPs) were not included to prevent non-evolutionary effects.

### Statistical analysis

The number (No.) and percentage (%) of the samples were presented as descriptive analyses. Wilson scores confidence interval method was used to measure the sensitivity, specificity, positive predictive value (PPV), and negative predictive value (NPV) of WGS after DST.

A *p*-value of <0.05 was considered significant. SPSS 21.0 (SPSS., USA) was employed for statistical analyses.

## Results

### Drug susceptibility profiles of R-R TB isolates

In total, 59 (8.38%) out of 704 clinical isolates collected between 1 January 2018 and 30 December 2019 were identified as RR-TB, including 83.05% MDR-TB, 27.12% pre-XDR, and 11.86% XDR (Table 1). Among the 59 RR-TB clinical isolates, 83.05, 100, 33.9, 64.41 13.56, 8.47, 38.98, 25.42, and 16.95% were resistant to INH, RIF, EMB, SM, AMK, CAP, LFX, PTO and PAS, respectively.

**Table 1:** Drug resistance profiles of RR-TB clinical samples (culture-based DST).

**Resistance pattern**	**No. (%) of isolates**
**Resistant to first-line drug**	
INH	49 (83.05)
RIF	59 (100.00)
EMB	20 (33.90)
SM	38 (64.41)
**Resistant to second-line drug**	
AMK	8 (13.56)
CAP	5 (8.47)
LFX	23 (38.98)
PTO	15 (25.42)
PAS	10 (16.95)
MDR	49 (83.05)
Pre-XDR	16 (27.12)
XDR	7 (11.86)

INH, isoniazid; RIF, rifampicin; EMB, ethambutol; SM, streptomycin; AMK, amikacin; CAP, capreomycin; LFX, levofloxacin; PTO, protionamide; PAS, para-amino salicylic acid; MDR, multidrug-resistant; pre-XDR, pre-extensively drug-resistant; XDR, extensively drug-resistant.

### Association of gene mutations with drug resistance

The results from the 59 RR-TB clinical isolates demonstrated the following: the genome 10 × coverage and genome 1 × coverage were from 51.67 to 238.11 (mean:166.29), from 98.69 to 99.95 (mean:99.19), and from 99.12 to 99.99 (mean:99.38).

The genes and mutations identified by WGS are linked to drug resistance (nine drugs) in the RR-TB isolates, which are listed in Supplemental Table 1.

Among the 59 RR-TB isolates, 13 (22.03%) strains had >1 mutations that are linked with INH resistance. The most common (n = 38) mutation was katG Ser315Thr and the second most common (n = 9) was fabG1 −8T>C.

Mutations linked with RIF resistance were detected in the 59 isolates (100.00%), and the rpoB gene had mutations in 100% of the samples. No other mutations were found in the isolates. All the isolates had mutations in the rpoB rifampicin resistance-determining region (RRDR, 81 bp). rpoB Ser531Leu (n = 37) was the most common.

The genes linked with resistance to SM were gid (n = 2), rspL (n = 33), and *rrs* (n = 2). Thirty-four strains showed EMB resistance. embA and embB mutations were found in two cases, and only onecase demonstrated mutations at two embB locations. The most common were embB Met306Val [52.94% (18/34)] and Met306Ile [23.53% (8/34)].

In respect to LFX, 23 (38.98%) isolates had gyrA gene mutations, of which the most common mutation observed was *gyrA* Asp94Gly [65.22% (15/23)]. Only one sample had gyrB mutation.

*rrs* gene mutations were found in all isolates that are resistant to AMK; 100% of the mutations were 1401a>g. *rrs* was found in genes linked to CAP resistance with the mutation 1401a>g (n = 6).

Fifteen strains showed PTO phenotype resistance, and 44 strains showed sensitivity. A total of 14 (93.33%) isolates had genetic mutations linked to PTO resistance. Mutations in both fabG1 (t-8c) and ethA genes (ethA 11a-ins, ethA 13a-ins, and ethA 11a-dup) were the most common (57.14%) (8/14).

Folate metabolism pathways, primarily thyA, folC, ribD, and dfrA, affect PAS resistance. Seven strains were resistant and demonstrated different mutations including folC Ile43Ser (n = 1), folC Ser150Gly (n = 1), folC Glu153Ala (n = 1), folC Glu153 Gly (n = 1), thyX(c−16t) (n = 1), and thyA Thr22Ala+ thyX(c−16t) (n = 2).

The phenotypic DST found resistance-related mutations in some of the samples. Mutations were discovered for INH at katG Ser315Gly (n = 1); for EMB at embB Met306Ile (n = 4), embB Met306Val (n = 6), embB Asp354Ala (n = 1), embB Gly406Asp (n = 2), and embB His1002Arg (n = 1); for CAP at *rrs* a1401g (n = 2); for LFX at gyrA Asp94Ala (n = 1); for PTO at fabG1 t-8c (n = 1), fabG1 c-15t (n = 1), ethA 403–424del (n = 1), ethA 61del (n = 1); for PAS at thyX c-16t (n = 1).

### Comparison of WGS and phenotypic DST results

The properties of each drug are listed in Table 2. A mean consistency of 93.79% (n = 59 isolates) was found for all the nie drugs, [76.27% (EMB) to 100% (RIF)]. The overall range of sensitivity and specificity for WGS were 70%−100% and 64.10%−100.00%, respectively. For both the highest sensitivity and specificity, 100% was found in 3 (RIF, EMB, and LFX) and 2 (SM and AMK) of the drugs respectively. The lowest sensitivity and specificity were found at 70% (PAS) and 64.1% (EMB), respectively. The phenotypic DST showed drug resistance in 10 samples (n = 2 for INH, n = 1 for SM, n = 2 for AMK, n = 1 for CAP, n = 1 for PTO, and n = 3 for PAS); however, the WGS results did not demonstrate any known drug-resistant mutations in the samples (Table 2).

**Table 2:** Whole-genome sequencing compared with phenotypic DST for detection of drug resistance in RR-TB.

**Drug**	**Phenotypically resistant**		**Phenotypically sensitive**		**Consistency (%)**	**Sensitivity^a^ (%)**	**Specificity^a^ (%)**	**PPV (%)**	**NPV (%)**
	**Genetically resistant**	**Genetically sensitive**	**Genetically resistant**	**Genetically sensitive**		**(95%CI, %)**	**(95%CI, %)**	**(95%CI, %)**	**(95%CI, %)**
INH	47	2	1	9	94.92	95.92 (84.86–99.29)	90.00 (54.11–99.48)	97.92 (87.53–99.89)	81.82 (47.76–96.79)
RIF	59	0	0	0	100.00	100.00 (92.38–100.00)	–	100.00 (92.38–100)	–
EMB	20	0	14	25	76.27	100.00 (79.95–100.00)	64.10 (47.15–78.32)	58.82 (40.83–74.87)	100.00 (83.42–100.00)
SM	37	1	0	21	98.31	97.37 (84.57–99.86)	100.00 (80.76–100.00)	100.00 (88.29–100.00)	95.45 (75.12–99.76)
AMK	6	2	0	51	96.61	75.00 (35.58–95.55)	100.00 (91.27–100.00)	100.00 (51.68–100.00)	96.23 (85.92–99.34)
CAP	4	1	2	52	94.92	80.00 (29.88–98.95)	96.30 (86.16–99.36)	66.67 (24.11–94.01)	98.11 (88.62–99.90)
LFX	23	0	1	35	98.31	100.00 (82.19–100.00)	97.22 (83.80–99.85)	95.83 (76.88–99.78)	100.00 (87.68–100.00)
PTO	14	1	4	40	91.53	93.33 (66.03–99.65)	90.91 (77.42–97.05)	77.78 (51.92–92.63)	97.56 (85.59–99.87)
PAS	7	3	1	48	93.22	70.00 (35.37–91.91)	97.96 (87.76–99.89)	87.5 (46.68–99.34)	94.12 (82.77–98.47)

a The sensitivity and specificity were examined with the Wilson score confidence interval method.

INH, isoniazid; RIF, rifampicin; EMB, ethambutol; SM, streptomycin; AMK, amikacin; CAP, capreomycin; LFX, levofloxacin; PTO, protionamide; PAS, para-amino salicylic acid; PPV, positive predictive value; NPV, negative predictive value; CI, confidence interval.

### Phylogenetic pattern of drug resistance

**Figure 1:**
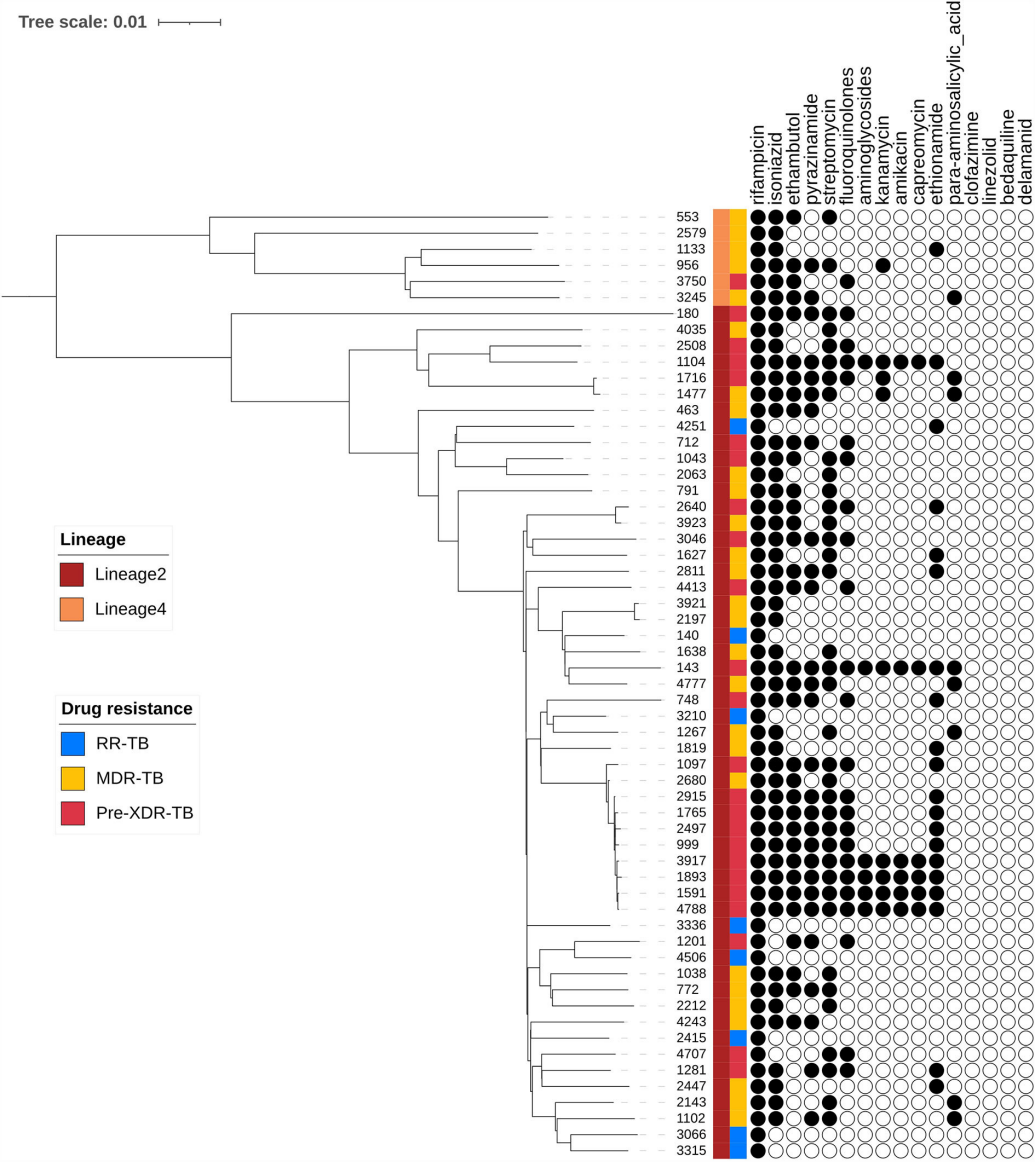
Phylogenetic tree of 59 R-R MTB clinical samples showing drug resistance profiles and lineages.

**Figure 2:**
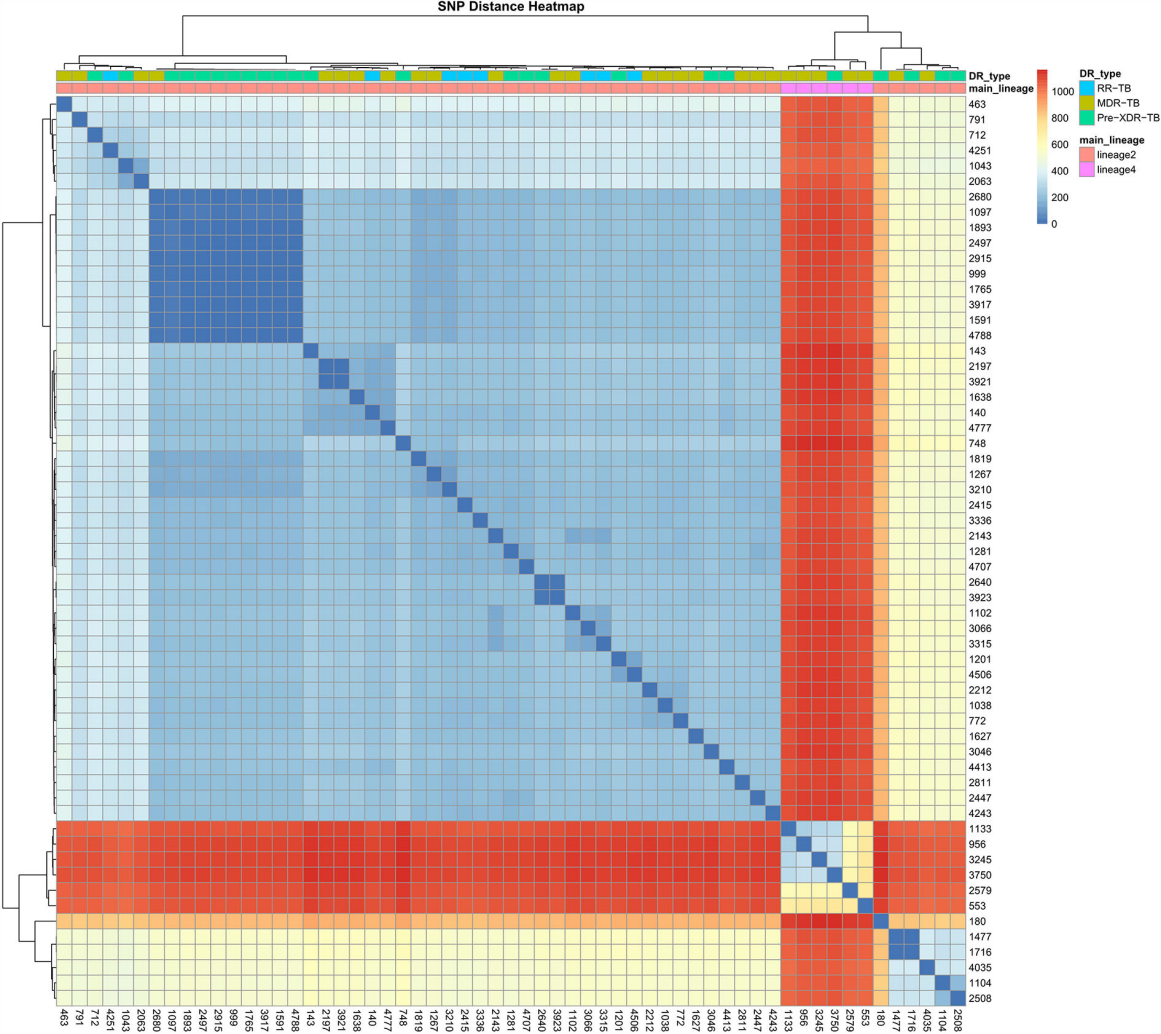
SNP difference heatmap of the 59 R-R MTB isolates.

A phylogenetic tree showing profiles and lineages related to drug resistance is shown in Figure 1. No transmission occurred among the 59 RR-TB strains, which is shown in Figure 2. Around 53 (89.83%) and 6 (10.17%) belonged to lineage two (East-Asian) and lineage four (Euro-American), respectively.

## Discussion

TB control and prevention, including diagnosis, treatment, and surveillance, can be guided by rapid, reliable, and increasingly affordable WGS technology. It effectively creates a genetic signature for drug resistance. Recently, WGS has been a critical player in predicting resistance to drugs (20–22). To the best of our knowledge, this study is the first to investigate gene mutations that impart resistance to nine anti-TB medications through the WGS method in Ningbo, China, and show that WGS is a reliable method for predicting resistance to INH, RIF, EMB, SM, AMK, CAP, LFX, PTO, and PAS with high accuracy, sensitivity, and specificity. The laboratories of Public Health England and the New York Department of Health have approved the use of WGS for testing susceptibility to TB drugs (23). Although WGS is widely conducted in research, its use in the clinical setting faces roadblocks since bioinformatics specialists are required for data analysis. Using raw sequences and the online software known as the TB-Profiler can predict drug resistance more accurately than other online platforms (24). In our study, WGS and the TB- Profiler were employed for prediction of drug resistance in 59 RR-TB clinical isolates. Mutations in *kat*G and *fab*G1 were discovered in 93.88% (46/49) of INH genotype-resistant isolates in our study, showing the role of the two locations in prediction of INH resistance. Ser315Thr was the most prevalent mutation in *kat*G; Ser315Thr previously has been shown to impart a significant level of INH resistance (25–27).

One previous review study showed that mutations in the 81-bp region of *rpo*B (RRDR) were responsible for more than 96% of RIF resistance (28), which was in line with our findings. Our study showed that no RIF-resistant bacteria were in this region. Outside the RRDR, mutations may not be detected by GeneXpert MTB/RIF (molecular test). Additionally, other mutations in the RRDR, such as a synonymous or silent mutations, may cause RIF resistance to be misdiagnosed (29).

In clinical isolates resistant to EMB, *emb*B codon 306 mutations were the most prevalent (30). The most common mutations in this analysis were *emb*B Met306Val and Met306Ile, which accounted for 52.94 and 23.53%, respectively.

Three resistance genes were found to be related to SM, notably *rrs, rps*L, and *gid*, which encode 16srRNA, ribosomal protein S12, and 16srRNA-specific methyltransferase, respectively (31, 32). Mutations in the *rps*L gene in our study, linked with high levels of resistance to SM, were predominant among SM-resistant isolates. We found that only isolates that did not have *rrs* or *rps*L mutations carried *gid* mutations. *gid* mutations linked with low resistance to SM were rare and only occurred in two (5.26%) SM genotype-resistant strains.

AMK and CAP genotype-resistant samples showed mutation 1401a>g in *rrs* linked to 70%−80% of MTB samples that are resistant to AMK and CAP globally (33).

Chromosome changes in the quinolone resistance determining region of *gyr*A or *gyr*B are the main way of fluoroquinolones resistance in MTB (34). Similar to previous studies (35, 36), the most prevalent mutations were found in positions 90 and 94 of *gyr*A in the LFX-resistant isolates, and lesser mutations were found in positions 46 and 91.

It is well known that the *inh*A c-15t mutation is associated with PTO resistance (37). We found that *fab*G1 t-8c and *eth*A genes mutation were most prevalent, and that no isolates harbored an *inh*A mutation in our study.

Regarding PAS, DST could not be successfully replaced by WGS in our study, but WGS may be more useful in better interpretation of gene mutations compared with DST. Despite high specificities, WGS failed to successfully diagnose PAS resistance because of the limited number of resistant strains.

The majority of drugs showed > 90% sensitivity and specificity, indicating that WGS is a reliable method for predicting drug resistance. The WGS results were consistent with the phenotypic DST for INH, RIF, SM, AMK, CAP, LFX, PTO, and PAS, showing that all were >90.00%, which was consistent with other studies with the same methods (38). A lower specificity for predicting EMB resistance than other drugs was found in our study. Around 35.9% of the EMB phenotype-sensitive isolates harbored mutations associated with EMB resistance. This could be because phenotypic DST for EMB is unreliable (39). However, it is unclear whether EMB resistance is affected by resistance mechanisms to other drugs (40).

The differences between WGS and phenotypic DST are explained by the following reasons. First, phenotypic DST may not catch samples that have low resistance to drugs. Recently, it was shown that phenotypic DST failed to diagnose EMB resistance, especially in the case of INH resistance (39, 40). Second, WGS may not be able to diagnose certain types of non-specific resistance, e.g., resistance that develops because of efflux pumps (41, 42).

Whole-genome sequencing has significant advantages over both phenotypic DST and molecular DST. WSG can detect specific genetic mutations promptly, which may be helpful for the most accurate treatment method. The detection capacity of WGS will also increase with time as novel genes involved in drug resistance are identified and the database is updated. Additionally, if certain laboratories do not have appropriate biosafety hoods, inactivation of strains may be performed and the inactivated strains may be sent to WGS labs, significantly reducing the time (months for DST vs. weeks for WGS). WGS will also become quicker and less expensive with the development of sequencing technology.

To our best knowledge, this was the first study on the agreement of phenotypic DST and WGS DST among rifampicin-resistant *Mycobacterium tuberculosis* (RR-TB) isolates in Ningbo. Our study may help in management of patients with TB and contribute to the development of novel anti-TB drugs and TB prevention strategies. Nonetheless, although the advanced sequencing technology of WGS and benefits were derived from WGS, some limitations of this study need to be considered. First, the study investigated a small number of anti-TB drugs, and pyrazinamide and linezolid were not included. Second, the number of amikacin-resistant isolates was small since AMK shows a low level of resistance. As a result, our results may be biased. Third, the small sample size number limited the statistical power of the study. There results must be interpreted with caution. However, our cohort represented the largest consecutive cohort of RR-TB in the region and the patients were representative thanks to the high detection rates of endemic TB. However, the findings of this study provided critical evidence for research and clinics to diagnose drug resistance by WGS and may help treat patients with high RR-TB burden.

## Data availability statement

The datasets presented in this study can be found in online repositories. The names of the repository/repositories and accession number(s) can be found at: http://www.ncbi.nlm.nih.gov/bioproject/862152, PRJNA862152.

## Ethics statement

Studies involving human participants were reviewed and approved by Ningbo CDC IBR and Hwa Mei Hospital IBR. Written informed consent for participation was not required for this study in accordance with the national legislation and institutional requirements.

## Author contributions

YC and YL conceived the study and were involved in the design, analysis, report writing, and drafting of the manuscript. TY, TC, GS, QC, and TH were involved in the conception and design, and supervised the study. QC and TH were involved in manuscript review. All authors contributed to the article and approved the submitted version.

## Funding

This study was funded by the Medical Technology Program Foundation of Zhejiang (Grant nos: 2021KY334 and 2022KY1189) and Ningbo Health Branding Subject Fund (Grant no: PPXK2018-10). The findings and conclusions of our study are not official representations of the Natural Science Foundation of Ningbo.

## Conflict of interest

The authors declare that the research was conducted in the absence of any commercial or financial relationships that could be construed as a potential conflict of interest.

## Publisher's note

All claims expressed in this article are solely those of the authors and do not necessarily represent those of their affiliated organizations, or those of the publisher, the editors and the reviewers. Any product that may be evaluated in this article, or claim that may be made by its manufacturer, is not guaranteed or endorsed by the publisher.
